# First Report of a Case of Pneumococcal Meningitis Which Did Not Respond to the Ceftriaxone Therapy despite the Isolated Organism Being Sensitive to This Antibiotic In Vitro

**DOI:** 10.1155/2011/485952

**Published:** 2011-10-25

**Authors:** Maryam Mojtabavi, Hamid Reza Naderi, Ashraf Tavanaee Sani, Mahmood Bagheri, Kiarash Ghazvini

**Affiliations:** ^1^Department of infectious disease, Faculty of Medicine, Mashhad University of Medical Sciences, Mashhad, Iran; ^2^Microbiology and Virology Research Center, Ghaem University Hospital and Faculty of Medicine, Mashhad University of Medical Sciences, Mashhad 9188974645, Iran

## Abstract

A 60-year-old man presented with pneumococcal meningitis which did not respond to the ceftriaxone therapy, in spite of in-vitro susceptibility (*minimal inhibitory concentration of 0.016 *μ*g/dLit*) of the isolated organism to this antibacterial agent, although ceftriaxone is still the drug of choice for such pneumococcal meningitis. Review of published articles revealed no report of clinical resistance in organisms which were susceptible to the same antimicrobial agent in vitro. This alarming emergence of isolates with in vivo resistance should be considered and even could lead to a shift in the empirical antibiotic therapy for pneumococcal infections.

## 1. Case Presentation

A 60-year-old man presented to the Emergency Department of Quaem Hospital, a university hospital in Mashhad, north-east of Iran, with seizures. The seizure occurred few hours before presentation and was associated with altered mental status and fever and a prodrome of headache. He had a history of epilepsy which began after a head trauma and fracture of skull in about 20 years ago. Seizures had been controlled with antiepileptic drugs and the therapy was discontinued few years later. 

At the time of presentation, the level of consciousness was decreased (Glasgow Coma Score of 7) and he had apparent neck stiffness accompanied by Kerning's and Brudzinski's sign on physical examination. The patient was febrile at the time of examination (oral temperature of 39°C) and other vital signs were as follows: systolic blood pressure of 160 mmHg, diastolic blood pressure of 90 mmHg, heart rate of 110 beats per minute, and respiratory rate of 16 per minute. The central and peripheral nervous systems were evaluated completely and no alteration or deficit was detected. There was no abnormal finding on assessment of the other organs. 

The empirical antimicrobial therapy (ceftriaxone 2 g every 12 hours, vancomycin 1 g every 12 hours, and ampicillin 2 g every 4 hours) in association with dexamethasone was begun after taking 2 blood samples for microbial culture. The patient was referred for brain CT scanning, and lumbar puncture had been done thereafter, when the imaging revealed neither contraindication for this procedure nor other intracranial complications. The cerebrospinal fluid had a cloudy appearance, polymorphonuclear pleocytosis (3440 white blood cell/*μ*L, with 92% polymorphonuclear cells), elevated protein levels (106 mg/dLit), and decreased glucose levels (2 mg/dLit). No organism observed on direct microscopy of CSF. 

In view of no significant improvement in the clinical condition of the patient, the second lumbar puncture had been performed 48 hours later and revealed no remarkable change in CSF parameters (2200 white blood cell/*μ*L with 90% polymorphonuclear cells, protein level of 220 mg/dLit, and glucose level of 12 mg/dLit); thus, the second brain CT scan with intravenous contrast was performed. There was hydrocephalus in lateral ventricles with increased density, suggesting intraventricular hemorrhage or debris as a complication of meningitis. 

No organism was isolated on either CSF cultures, but small colonies of *Streptococcus pneumoniae* with *α* hemolysis appeared on both blood cultures obtained at the time of admission. Antibiotic susceptibility was performed for the isolated organism and MIC of ceftriaxone against this strain was estimated by *E*-test. According to the MIC result, the organism was susceptible to ceftriaxone (MIC = 0.016 *μ*g/dLit); hence, other antimicrobials (including ampicillin and vancomycin) were discontinued 3 days after admission. 

Despite adequate antimicrobial therapy, the fever persisted and clinical situation of patient continued worsening and he experienced respiratory failure which required endotracheal intubation and mechanical ventilation on 5th day; his condition deteriorated continuously and was complicated by gastrointestinal bleeding and pleural effusion appeared as well. 

Patient clinical state and signs of ongoing infection implied the possibility of a resistant pneumococcus; then rifampin was added to the antimicrobial regimen (on 10th day after admission), which resulted in significant clinical improvement. The patient defervesced, his level of consciousness increased to near normal status, the respiration got better, and he was extubated 3 days later.

## 2. Discussion

*Streptococcus pneumoniae* is the most common cause of acute bacterial meningitis in adults, and even with appropriate antimicrobial therapy, case-fatality rates for high-risk patients can be as high as 55% for meningitis [1–4]. An alarming increase of isolates with antimicrobial resistance has been noted [5–9], leading to shifts in the empirical antibiotic therapy for pneumococcal infections [10, 11]. Currently, ceftriaxone or cefotaxime are the drugs of choice for pneumococcal meningitis caused by a penicillin nonsusceptible strain [12, 13]. As treatment failure of therapeutic regimens such as penicillin, chloramphenicol, third-generation cephalosporins, and vancomycin has been frequently reported [14, 15], susceptibility against antibiotics especially ceftriaxone should be evaluated according to guidelines established by the NCCLS [16]. *S. pneumoniae* strains are considered sensitive if the MIC of ceftriaxone is lower than or equal to 1 *μ*g/dLit; intermediately resistant for MICs of 1 to 2 *μ*g/dLit; highly resistant for MICs equal to or higher than 4 *μ*g/dLit. 

The bacteriological failure of cefazolin, a first-generation cephalosporin as well as the second-generation cephalosporin, cefuroxime, and the poorly active third-generation cephalosporin, and ceftazidime, have all been described previously [17–19]. There is a single case report of a bacteriological failure of low-dose cefotaxime (100 mg/kg/day) plus gentamicin in a patient with a bacteremia caused by a highly resistant (cefotaxime MIC of 6 mg/mL) pneumococcus [20]. This patient developed meningitis and the failure may have been due to seeding of organisms from the meninges to the blood. 

There was only a few studies that reported conflicting results including data regarding the impact of discordant antibiotic therapy (i.e., antimicrobial agents to which strains are resistant in vitro) on the outcome of pneumococcal pneumonia. Although therapeutic failures has been documented when ceftriaxone has been used for meningitis caused by ceftriaxone-resistant strains (MIC ≥ 2 g/mL) [14, 21, 22], especially if ceftriaxone monotherapy was used [23]; a review of published articles revealed no report of clinical resistance with organisms which in vitro were susceptible to this agent. This patient is the first case of in-vitro sensitive pneumococcal meningitis which did not respond to the ceftriaxone therapy, and even to the combination regimen of ceftriaxone and vancomycin. Adding the rifampin to the therapeutic regimen led to the significant clinical improvement. In cases of meningitis caused by rifampin-susceptible strain, the combination of ceftriaxone and rifampin showed a much higher bactericidal activity than that of ceftriaxone and vancomycin or meropenem plus vancomycin [23]. Animal models of meningitis have demonstrated activity of rifampin paired with one of these agents, even with cephalosporin-resistant organisms [24]. Therefore, it is suggested that rifampin should be used in combination if the organism is susceptible to rifampin or if there is a delay in the treatment response to regimens without rifampin [23, 25].
